# Homeodynamic Rejuvenation: an adaptive framework for resetting skin aging

**DOI:** 10.3389/fragi.2026.1840035

**Published:** 2026-06-03

**Authors:** Anthony Brown, Carla Simonetto, Carlos López-Otín

**Affiliations:** 1 Innovation and Development, ISDIN, Barcelona, Spain; 2 Centre de Recherche des Cordeliers, Equipe Labellisée par la Ligue Contre le Cancer, Inserm U1138, Université Paris Cité, Sorbonne Université, Paris, France; 3 Facultad de Ciencias de la Vida y la Naturaleza, Universidad Nebrija, Madrid, Spain

**Keywords:** adaptive capacity, photoaging, exposome, extracellular matrix, homeodynamic plasticity, Homeodynamic Rejuvenation, homeodynamics, skin aging

## Abstract

Skin aging reflects not only the accumulation of molecular damage, but also a progressive decline in the skin’s ability to restore equilibrium under continuous environmental stress. Classical models distinguishing intrinsic and extrinsic aging do not fully capture this dynamic process. In this article, we introduce Homeodynamic Rejuvenation as the restoration of functional competence and biological vitality, achieved not through reversal of visible signs of aging, but by re-establishing the skin’s ability to detect stress, coordinate adaptive responses, and recover efficiently following perturbation. Central to this framework is the concept of homeodynamic plasticity, which reflects the skin’s intrinsic capacity to dynamically sustain function under environmental and metabolic stress. By integrating principles of homeodynamics with exposome biology, this approach targets the reactivation of the cellular systems that govern stress sensing, repair, and recovery. Five core biological processes, namely, intracellular quality control, regenerative competence, metabolic resilience, cellular integrity, and structural integrity, underpin homeodynamic plasticity. Disruption or failure of one or more of these processes results in progressive functional decline, culminating in skin aging, of which dermatoporosis, a chronic cutaneous insufficiency syndrome, represents a terminal manifestation. These interconnected processes provide a coordinated basis for both restoring and assessing skin function. We propose that Homeodynamic Rejuvenation is best evaluated through dynamic perturbation–recovery kinetics, which quantify the skin’s ability to respond to and recover from stress. By linking these biological processes to measurable functional and clinical outcomes, Homeodynamic Rejuvenation offers a structured and translational framework for quantifying and restoring skin resilience.

## 1 Introduction

### 1.1 Conceptual models of skin aging

The visible manifestations of skin aging, such as wrinkling, laxity, dyspigmentation, dryness, and reduced elasticity, arise from progressive alterations in cellular function, extracellular matrix (ECM) organization, and tissue repair capacity. Early conceptual models of skin aging (Montagna and Carlisle, 1979; Gilchrest, 1989) described these changes as the consequence of intrinsic, genetically programmed declines in regenerative activity and repair mechanisms (Harman, 1956; Harley et al., 1990; Balaban et al., 2005; Kaushik and Cuervo, 2015), processes that are further accelerated by environmental and lifestyle stressors (Krutmann et al., 2017; 2021).

Subsequent frameworks expanded this mechanistic understanding. The Hallmarks of Aging (López-Otín et al., 2013) identified a set of conserved biological processes that drive age-related decline, including genomic instability, telomere attrition, epigenetic alterations, loss of proteostasis, deregulated nutrient sensing, mitochondrial dysfunction, cellular senescence, stem cell exhaustion, and altered intercellular communication. Later refinements incorporated additional contributors, such as chronic inflammation, defective autophagy, and microbiome dysbiosis (López-Otín et al., 2023). Together, these hallmarks describe how progressive impairment of maintenance systems leads to functional deterioration across tissues.

In parallel, the concept of the skin exposome emphasized the dominant influence of environmental and lifestyle factors, including ultraviolet (UV) radiation, pollution, climate, and behavioral stressors, in shaping cutaneous aging (Krutmann et al., 2017). It is widely estimated that approximately 80% of visible skin aging results from extrinsic exposomic influences, while only approximately 20% arises from intrinsic chronological processes (Krutmann et al., 2017; 2021).

### 1.2 Skin aging and adaptive capacity

Although these models have substantially advanced mechanistic understanding, they remain largely descriptive in explaining how aging manifests in exposed tissues such as skin. The skin does not passively accumulate damage in proportion to exposure; rather, it continuously senses environmental perturbations and engages dynamic repair and adaptive responses to restore equilibrium. Consequently, individual variation in skin aging is determined less by exposure alone than by the efficiency with which the skin detects stress, activates repair pathways, and re-establishes functional balance. Skin aging can thus be considered a progressive loss of adaptive capacity, in which maintaining vitality depends on preserving the mechanisms of adjustment and recovery that sustain functional balance (Ukraintseva et al., 2021). Consistent with this view, longitudinal multi-omics studies have demonstrated that skin aging does not follow a linear trajectory, but instead progresses through non-linear kinetics, characterized by extended periods of relative stability punctuated by tipping points that precipitate rapid functional decline (Lehallier et al., 2019; Shen et al., 2024). These transitions exemplify the progressive erosion of adaptive capacity, marking the point at which compensatory and recovery mechanisms become insufficient to maintain homeostasis, thereby triggering a shift toward accelerated deterioration.

## 2 The skin is a homeodynamic organ

Unlike internal organs that operate within relatively stable physiological environments, the skin functions at the interface between the organism and the external world. It is, therefore, continuously exposed to fluctuations in UV radiation, temperature, humidity, mechanical stress, pollution, and microbial challenges. Under these conditions, the skin does not exist in a static state of homeostasis, but instead continuously senses, responds to, and recovers from environmental perturbations. In this context, equilibrium is not defined by the maintenance of a fixed set point but rather emerges from adaptive interactions among its components (Lloyd et al., 2001; López-Otín and Kroemer, 2021). This dynamic state is referred to as homeodynamics.

**FIGURE 1:**
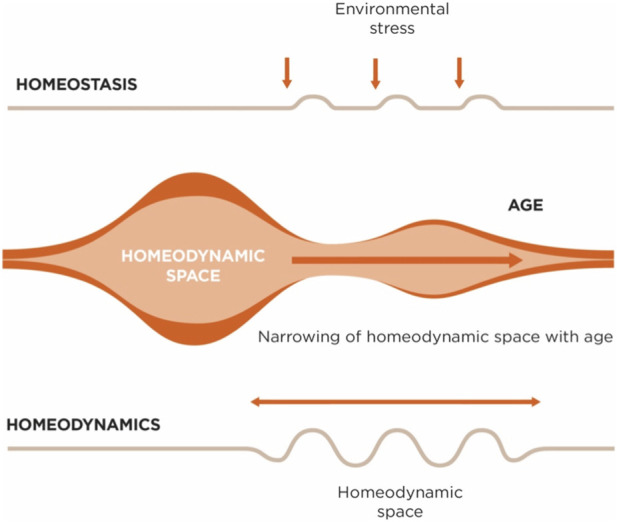
Conceptual illustration of homeodynamics versus homeostasis in skin aging. In homeostasis (top), stability is maintained through limited correction of small perturbations caused by environmental stress. In homeodynamics (middle and bottom), stability is achieved within a flexible “homeodynamic space,” which expands and contracts in response to environmental challenges. This adaptive range allows the skin to maintain function through continual adjustment and recovery. Over time, with cumulative stress and aging, this homeodynamic space gradually narrows, and the skin’s homeodynamic plasticity can become insufficient to restore function after perturbation.

The concept of homeodynamics was first introduced by Yates (1994) and later elaborated by Rattan (2007) to describe biological systems not as static entities maintaining fixed set points, but rather as dynamic systems characterized by continuous adaptation. Central to this framework is the notion of a homeodynamic space—a flexible physiological range within which cells, tissues, or organisms can respond to environmental and metabolic perturbations while preserving functional integrity (Figure 1) (Rattan, 2007). Rather than maintaining strict equilibrium, biological systems operate within this adaptable range, constantly adjusting to internal and external stressors.

In the context of skin biology, the size and integrity of the homeodynamic space fundamentally govern its capacity to respond to chronic environmental challenges, including UV radiation, visible light, pollution, and oxidative stress. A larger homeodynamic space reflects a broader adaptive range over which the skin can sense perturbations, mount coordinated responses, and recover efficiently. We propose that this capacity of the skin to dynamically adjust, reorganize, and sustain function in response to stress be termed *homeodynamic plasticity*.

Cumulative molecular damage, impaired repair mechanisms, cellular senescence, and dysregulated antioxidant defenses progressively reduce homeodynamic plasticity. As it declines, the skin’s ability to detect perturbations, coordinate responses, and recover efficiently becomes compromised. This loss of plasticity impairs efficient repair, regeneration, and the maintenance of barrier and structural integrity, rendering the skin increasingly vulnerable to perturbations and shifting it from reversible dysfunction to persistent damage.

Accordingly, this perspective shifts the focus of intervention from simply preventing or masking damage to restoring the skin’s homeodynamic plasticity and, consequently, its effective adaptive range.

## 3 Homeodynamic Rejuvenation

This leads us to propose the concept of *Homeodynamic Rejuvenation*. Homeodynamic Rejuvenation integrates two complementary principles: homeodynamics, which captures the skin’s intrinsic homeodynamic plasticity, i.e., its capacity to dynamically adapt, repair, and rebalance in response to continuous environmental and metabolic stress; and rejuvenation, defined not as the reversal of visible signs of aging but as the restoration of functional competence and biological vitality.

Within this framework, aging reflects a progressive loss of homeodynamic plasticity, while rejuvenation corresponds to its restoration. The goal is, therefore, to re-establish the skin’s ability to detect stress, mount an appropriate response, and recover efficiently following perturbation.

### 3.1 Positioning Homeodynamic Rejuvenation within the Hallmarks of Health and Aging

Unlike existing models, such as the Hallmarks of Health (López-Otín and Kroemer, 2021) and the Hallmarks of Aging (López-Otín et al., 2013; 2023), both co-developed by one of the authors, which conceptualize resilience and define the mechanisms that undermine it, respectively, Homeodynamic Rejuvenation is proposed as a bridge between these frameworks, translating their system-level and mechanistic insights into a structured, skin-specific approach.

The Hallmarks of Health framework defines health as a dynamic, actively maintained biological state rather than the mere absence of disease (López-Otín and Kroemer, 2021). Within this model, biological function emerges from coordinated processes spanning multiple levels of complexity, organized into three overarching domains: spatial compartmentalization, maintenance over time, and adaptive responses to stress (López-Otín and Kroemer, 2021). Central to this framework is adaptive capacity, sustained by the integrated action of homeostatic resilience, hormetic regulation, and repair and regeneration (López-Otín and Kroemer, 2021). Together, these processes enable biological systems to absorb perturbations, mount adaptive responses, and restore function following damage.

**FIGURE 2:**
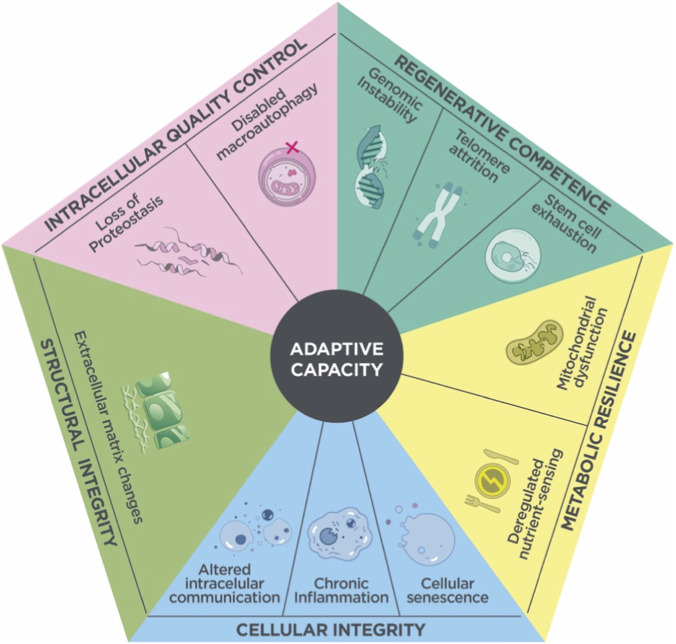
Integration of hallmarks of aging into core domains underpinning homeodynamic plasticity. Classical hallmarks of aging are integrated into five functional domains that support the skin’s adaptive capacity: intracellular quality control, regenerative competence, metabolic resilience, cellular integrity, and structural integrity. Each domain groups mechanistically related processes (e.g., proteostasis and autophagy; genomic instability and stem cell exhaustion; mitochondrial dysfunction; senescence and inflammation; ECM alterations). This integration highlights how diverse molecular drivers converge onto a limited set of systems governing the skin’s ability to respond to stress and maintain functional equilibrium.

Complementing this, the Hallmarks of Aging describe the molecular and cellular mechanisms that progressively impair its capacity to do this, including genomic instability, loss of proteostasis, mitochondrial dysfunction, cellular senescence, and altered intercellular communication (López-Otín et al., 2013; 2023). These hallmarks act in coordination, but how their combined actions are organized remains poorly defined. In the skin, we propose that these mechanisms can be organized into a limited set of processes that underpin adaptive capacity: intracellular quality control, regenerative competence, metabolic resilience, cellular integrity, and structural integrity (Table 1; Figure 2). We also propose that these domains represent core systems through which molecular damage is processed, compensated for, or propagated, thereby linking upstream perturbations to downstream functional decline.

**TABLE 1:** Functional domains supporting homeodynamic plasticity in the skin.

Domain	Integrated hallmark	Core biological role	Dominant cellular program	Key molecular driver	Tissue-level consequence
Intracellular quality control	Loss of proteostasis; disabled macroautophagy	Maintains proteome integrity, organelle turnover, and detoxification capacity	Impaired autophagy; accumulation of damaged proteins and organelles	ROS/RNS; glycoxidative stress; impaired clearance systems	Cellular dysfunction; increased oxidative burden; sensitization to stress
Regenerative competence	Genomic instability; telomere attrition; stem cell exhaustion	Preserves capacity for repair, renewal, and tissue regeneration	Replicative decline; impaired stem cell function; reduced proliferation	DNA damage; telomere shortening; epigenetic drift	Epidermal thinning; delayed repair; reduced turnover
Metabolic resilience	Mitochondrial dysfunction; deregulated nutrient sensing	Sustains ATP production, redox balance, and cellular adaptability	Mitochondrial dysfunction; reduced ATP; increased ROS production	Oxidative stress; mitochondrial damage; nutrient signaling imbalance (mTOR/AMPK)	Energy deficit; impaired repair capacity; amplification of oxidative stress
Cellular integrity	Cellular senescence; altered intracellular communication; chronic inflammation	Maintains controlled signaling networks and coordinated stress responses	Senescence accumulation; SASP; chronic inflammation	NF-κB and AP-1 activation; cytokine signaling	ECM degradation; chronic inflammation; disrupted regeneration
Structural integrity	ECM dysfunction	Maintains mechanical resilience and cell-matrix signaling	ECM fragmentation; impaired mechanotransduction	MMP induction; reduced collagen synthesis; glycation crosslinking	Wrinkling; dermal atrophy; loss of elasticity and firmness

Abbreviations: ROS, reactive oxygen species; RNS, reactive nitrogen species; ATP, adenosine triphosphate; mTOR: mechanistic target of rapamycin; AMPK, AMP-activated protein kinase; SASP, senescence-associated secretory phenotype; NF-κB, nuclear factor kappa B; AP-1, activator protein 1; ECM, extracellular matrix; MMP, matrix metalloproteinase.

Hallmarks such as loss of proteostasis and impaired macroautophagy are incorporated into intracellular quality control as they collectively maintain proteome integrity, organelle turnover, and redox balance. Genomic instability, telomere attrition, and stem cell exhaustion are aligned within regenerative competence, reflecting their shared role in preserving the capacity for repair, renewal, and tissue regeneration. Mitochondrial dysfunction and deregulated nutrient sensing are grouped within metabolic resilience, given their central role in sustaining energy homeostasis, redox equilibrium, and cellular adaptability. Altered intercellular communication, chronic inflammation, and cellular senescence are integrated within cellular integrity, capturing the regulation of signaling networks that coordinate local and systemic responses to stress. Within this domain, the accumulation of senescent cells and their associated secretory phenotype (SASP) represents a central convergence point that amplifies inflammation, disrupts regenerative processes, and promotes ECM degradation (Basisty et al., 2020).

Finally, processes governing ECM composition and remodeling are grouped within structural integrity, reflecting their essential role in maintaining mechanical resilience and mediating bidirectional signaling between cells and their microenvironment.

### 3.2 An integrated model of homeodynamic plasticity in the skin

**FIGURE 3:**
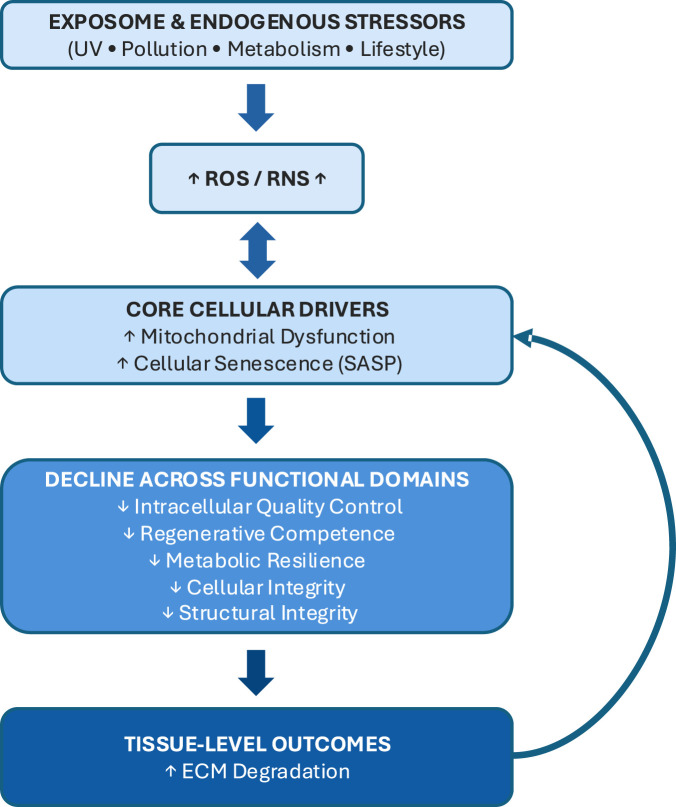
Convergent model of the drivers underlying failure of homeodynamic plasticity in skin aging. Reactive oxygen and nitrogen species (ROS/RNS), generated through intrinsic metabolism and environmental stressors, act as key upstream drivers of dysfunction. These signals converge to promote mitochondrial dysfunction and cellular senescence, which together reduce metabolic capacity and promote chronic inflammatory signaling. This convergence impairs core adaptive functions, including regenerative competence, metabolic resilience, and cellular integrity. The resulting loss of homeodynamic plasticity drives extracellular matrix (ECM) deterioration and loss of structural integrity, which define the functional and visible manifestations of skin aging. Feedback from ECM disruption further reinforces mitochondrial dysfunction and senescence, establishing a self-amplifying cycle of progressive decline.

Building on this domain-based organization, skin aging can be understood as the progressive loss of homeodynamic plasticity driven by the failure of these core adaptive functions under continuous environmental exposure. Reactive oxygen and nitrogen species, generated both endogenously during metabolism and exogenously in response to stressors such as ultraviolet radiation and pollution, act as key upstream drivers of this process (Figure 3).

Crucially, within this interconnected framework, disruption in one domain does not remain isolated but instead propagates dysfunction across the system. Mitochondrial impairment, for example, reduces ATP availability required for repair while increasing reactive oxygen species (ROS) production, thereby reinforcing oxidative stress and metabolic dysregulation (Balaban et al., 2005; Rinnerthaler et al., 2015; Sun et al., 2016; Stout and Birch-Machin, 2019). Similarly, the accumulation of senescent cells and their SASP amplifies chronic inflammation and promotes widespread cellular dysfunction (Dimri et al., 1995; Campisi, 2013; Basisty et al., 2020).

These changes ultimately converge at the tissue level, where structural integrity is progressively compromised. Notably, ECM alterations, now recognized as a standalone hallmark of aging (Kroemer et al., 2025), not only compromise mechanical integrity but also perpetuate cellular dysfunction through mechanotransductive and metabolic feedback. Disruption of collagen and elastin homeostasis compromises dermal structure and mechanical resilience (Seo et al., 2001; Monnier et al., 2005; Fisher et al., 2014; Liu et al., 2024; Paculová et al., 2025) while simultaneously feeding back to influence mitochondrial function, senescence programs, and stem cell niche dynamics (Saraswathibhatla et al., 2023; Schnellmann and Gerecht, 2023; Zhang et al., 2024).

Thus, degradation of the ECM illustrates the interconnected nature of cutaneous adaptive failure: disruption in any single domain, such as impaired repair leading to increased senescence and reduced energy availability, can initiate a cascade of dysfunction that progressively undermines the skin’s ability to adapt and recover. Dermatoporosis, a chronic cutaneous insufficiency syndrome (Kaya and Saurat, 2007; Kaya and Saurat, 2010), exemplifies this.

### 3.3 Dermatoporosis as a paradigm of failure of homeodynamic plasticity

At the tissue level, dermatoporosis is defined by severe ECM degradation, including fragmentation of collagen fibers, loss of elastin integrity, and basement membrane disruption, leading to reduced mechanical resilience and increased susceptibility to tearing and deep dissecting hematomas (Kaya and Saurat, 2007; Kaya et al., 2019). These changes are accompanied by impaired repair and renewal, as reflected in delayed wound healing and reduced regenerative capacity (Kaya and Saurat, 2010).

At the cellular level, dermatoporotic skin exhibits features consistent with metabolic decline and impaired stress responses. Studies have shown the accumulation of senescent fibroblasts and increased expression of senescence markers, contributing to chronic inflammation and matrix degradation (Barnes et al., 2017; Kaya et al., 2023; Widgerow et al., 2024). Although direct studies of mitochondrial function in dermatoporosis remain limited, aged and fragile skin shows mitochondrial dysfunction, reduced ATP production, and increased oxidative stress, all of which compromise the energy supply required for repair processes (Rinnerthaler et al., 2015; Stout and Birch-Machin, 2019).

Dermatoporosis can thus be conceived as arising when compensatory mechanisms are no longer sufficient to maintain homeostasis. Beyond this threshold, the process becomes self-reinforcing as failure within one domain propagates across others, most notably through feedback loops linking mitochondrial dysfunction, cellular senescence, and ECM degradation.

Dermatoporosis, however, appears to retain a degree of reversibility. Kaya et al. (2023) demonstrated that topical treatment with a cream containing 0.05% retinaldehyde and 1% intermediate-size hyaluronate fragments, applied twice daily for 30 days, significantly reduced the number of p16Ink4a-positive senescent cells in both the epidermis and dermis of patients with dermatoporosis, accompanied by a significant clinical improvement. These findings support the concept that targeting key drivers of adaptive failure may expand residual homeodynamic plasticity and improve clinical outcomes.

### 3.4 Translational implications of Homeodynamic Rejuvenation

Accordingly, Homeodynamic Rejuvenation is designed to enable both the targeted modulation of the skin’s homeodynamic plasticity and the comprehensive evaluation of its functional impact. Crucially, it is not inherently linked to any type of specific intervention but is designed to act as a unifying biological framework applicable to topical, systemic, and procedural strategies (Table 2).

**TABLE 2:** Mapping of selected interventions across skin adaptive functions.

Domain	Specific hallmark/process	Topical intervention	Oral/Systemic intervention	Procedural/Interventional
Intracellular quality control	Loss of proteostasis; impaired macroautophagy; redox imbalance	Retinoids; niacinamide Nrf2 activators	Metformin; rapamycin; polyphenols; spermidine; urolithin A; resveratrol	PBM; microneedling
Regenerative competence	Genomic instability; telomere attrition; stem cell exhaustion	DNA repair enzymes (e.g., photolyase); niacinamide; retinoids; growth-factor-based therapies	Nicotinamide; hormonal modulation; NAD^+^ precursors (NR and NMN)	Fractional laser; microneedling; PDT; PRP
Metabolic resilience	Mitochondrial dysfunction; deregulated nutrient sensing	Niacinamide; CoQ10; vitamin C; melatonin	Metformin; rapamycin; carotenoids; NAD^+^ precursors (NR and NMN); acarbose; resveratrol; urolithin A	PBM; RF; HIFU
Cellular integrity	Altered intercellular communication; chronic inflammation; cellular senescence (SASP)	Niacinamide; polyphenols; melatonin; exosome-based therapies	Nicotinamide; polyphenols; senotherapeutics (dasatinib + quercetin; fisetin; navitoclax)	PDT; laser; RF
Structural integrity	ECM degradation; impaired remodeling; loss of mechanical resilience	Retinoids; vitamin C; peptides	Collagen peptides; HA; isotretinoin; hormonal modulation	Laser resurfacing; RF; microneedling; HIFU

Abbreviations: CoQ10, coenzyme Q10; ECM, extracellular matrix; HA, hyaluronic acid; HIFU, high-intensity focused ultrasound; NAD^+^, nicotinamide adenine dinucleotide; NMN, nicotinamide mononucleotide; Nrf2, nuclear factor erythroid 2-related factor 2; NR, nicotinamide riboside; PBM, photobiomodulation; PDT, photodynamic therapy; PRP, platelet-rich plasma; RF, radiofrequency; SASP, senescence-associated secretory phenotype.

Its translational value lies in its ability to identify interventions, particularly combinations, which produce coordinated improvements across multiple domains and to determine whether such effects translate into enhanced adaptive capacity and durable clinical benefit. Interventions that act across several adaptive processes should be prioritized, as targeting a single pathway in isolation may result in limited efficacy or unintended imbalance. In contrast, pleiotropic strategies, whether through individual agents or rational combinations, are more likely to elicit coordinated biological responses, supporting adaptation while preserving system-level balance.

Topical therapies remain foundational in this framework, and many commonly used cosmetic ingredients act on key biological processes that underpin skin homeodynamic plasticity. Niacinamide (nicotinamide), for example, exhibits antioxidant activity, enhances barrier function, reduces inflammation, and supports DNA repair through its role in NAD^+^ metabolism (Gehring, 2004). Retinoids regulate keratinocyte differentiation, stimulate collagen synthesis, and suppress matrix metalloproteinases (Fisher et al., 1996; Kafi et al., 2007), while peptides derived from elastin, connective tissue glycoproteins, or collagens stimulate the deposition of ECM components (Maquart et al., 2004). Similarly, vitamin C contributes to antioxidant defense while also participating in collagen synthesis (Pullar et al., 2017), whereas coenzyme Q10 and melatonin act as antioxidants and modulators of mitochondrial function (Fischer and Elsner, 2001; Bentinger et al., 2010; Knott et al., 2015; Reiter et al., 2016; Tan et al., 2016; Slominski et al., 2018; Holtkamp et al., 2023).

Finally, biologic approaches, such as growth factors, platelet-rich plasma, and exosomes, aim to enhance regenerative signaling and tissue remodeling in photoaged skin (Fitzpatrick and Rostan, 2003; Werner and Grose, 2003; Alves and Grimalt, 2018; Kalluri and LeBleu, 2020; Asubiaro and Avajah, 2024).

This principle also extends to systemic therapies. Oral isotretinoin, for example, has been shown to induce coordinated dermal remodeling in photoaged skin (Rabello-Fonseca et al., 2009), while supplementation with hydrolyzed collagen and hyaluronic acid has been associated with improvements in skin barrier function, ECM quality, and hydration status (de Miranda et al., 2021; Michelotti et al., 2021). Oral photoprotective agents such as nicotinamide, *Polypodium leucotomos* extract, and carotenoids have also been shown to exert broad effects, including reducing oxidative stress, DNA damage, and immunosuppression (Natarelli et al., 2025). Hormonal modulation (e.g., estrogen replacement therapy) is also known to influence dermal structure and function (Brincat et al., 1987).

Systemic agents widely employed in geromedicine research have also shown significant promise. Although direct clinical evidence in skin remains limited for many of these compounds, their effects on the core processes governing homeodynamic plasticity, together with supporting preclinical and emerging human data, suggest meaningful translational potential. Metformin, for example, activates AMPK and inhibits mTOR signaling, thereby influencing cellular energetics, inflammation, and repair processes (Barzilai et al., 2016). It has demonstrated anti-inflammatory and matrix-protective effects in skin models, including reductions in NF-κB activity and MMP expression in human foreskin fibroblasts and mouse skin (Chen et al., 2022). Rapamycin reduces markers of cellular senescence and improves dermal matrix organization in photoaged human skin (Chung et al., 2019). Caloric restriction mimetics such as resveratrol and acarbose induce antioxidant and matrix-protective responses, attenuating UV-induced damage and supporting collagen homeostasis (Baur et al., 2006; Harrison et al., 2014). NAD^+^ precursors, including nicotinamide riboside (NR) and nicotinamide mononucleotide (NMN), enhance mitochondrial energetics, DNA repair, and stress-response pathways, with preclinical evidence indicating improved resistance to UV-induced damage and enhanced cellular repair capacity in the skin (Yoshino et al., 2018). Compounds such as spermidine and urolithin A promote intracellular quality control through the induction of autophagy and mitophagy, while spermidine directly modulates epithelial stem cell function (Ramot et al., 2011; Ryu et al., 2016; Hofer et al., 2024). Finally, senolytic agents, including dasatinib plus quercetin (D + Q), fisetin, and navitoclax, enhance cellular integrity by selectively eliminating senescent cells and attenuating SASP-mediated inflammation (Kirkland and Tchkonia, 2020).

Procedural interventions provide a complementary dimension by directly inducing controlled tissue remodeling. Laser therapies (e.g., fractional CO_2_ and erbium:YAG), radiofrequency devices, and microneedling stimulate collagen production and ECM reorganization through the activation of wound-healing pathways (Hantash et al., 2006; Aust et al., 2008; Fisher et al., 2008; Elsaie, 2009). Ultrasound-based technologies (e.g., high-intensity focused ultrasound) similarly induce dermal remodeling through the thermal and mechanical stimulation of collagen contraction and neocollagenesis (Alam et al., 2010). Photodynamic therapy targets damaged or precancerous cells while improving signs of photoaging (Babilas et al., 2010). In parallel, photobiomodulation (PBM) using low-level red or near-infrared light (≈600–900 nm), enhances mitochondrial activity via cytochrome c oxidase, increases ATP production, and modulates redox signaling, promoting collagen synthesis and reducing inflammation (Hamblin, 2017).

Notably, many of these strategies function, at least in part, through hormesis, triggering adaptive stress responses that enhance repair, regeneration, and resilience (López-Otín and Kroemer, 2021). Retinoids induce mild irritation that stimulates renewal; laser and microneedling create controlled micro-injury that activates repair cascades (Fisher et al., 2002); PBM delivers sub-thermal photonic stress that activates mitochondria (Hamblin, 2017); and phytochemicals, such as resveratrol, stimulate endogenous defense systems (Schäfer and Werner, 2015; Kim et al., 2018). Even systemic agents such as metformin and rapamycin can be interpreted in this way as they impose metabolic constraints that promote adaptive cellular responses (Zhou et al., 2001; Johnson et al., 2013; Saxton and Sabatini, 2017; Barzilai et al., 2018).

### 3.5 Biological constraints and translational considerations

Modulation of core aging pathways, however, must also account for inherent biological trade-offs (Kirkwood, 1977; Kirkwood and Austad, 2000). Excessive stimulation of ECM synthesis, for example, may promote fibrotic remodeling through dysregulated TGF-β signaling and collagen deposition (Wynn and Ramalingam, 2012). Similarly, although clearance of senescent cells has shown promise in preclinical models, senescent cells can also contribute to tissue repair and wound healing, and their indiscriminate removal may impair regenerative processes (Demaria et al., 2014; Kirkland and Tchkonia, 2017). Enhancement of mitochondrial activity similarly presents a context-dependent effect, as increased oxidative phosphorylation may elevate ROS production if not balanced by adequate antioxidant defenses (Ristow and Schmeisser, 2014).

Consequently, effective intervention strategies should aim not for maximal activation of individual pathways, but rather for their coordinated modulation within physiological limits. Within the Homeodynamic Rejuvenation framework, this implies maintaining a functional balance across domains, where enhancing one process does not come at the expense of another but instead contributes to an overall improvement in adaptive function.

### 3.6 Dynamic assessment through perturbation–recovery modeling

The shift in therapeutic paradigms introduced by Homeodynamic Rejuvenation, from correcting visible signs of aging to addressing their root cause, has direct implications for how efficacy is defined and measured. Traditional endpoints, focused on visible or structural outcomes such as wrinkle depth, pigmentation, or elasticity, primarily capture downstream manifestations of aging rather than the skin’s underlying ability to respond to stress. Within a homeodynamic framework, greater emphasis should, therefore, be placed on functional and adaptive biomarkers, which more directly reflect the restoration of resilience and may provide earlier and more mechanistically meaningful indicators of intervention efficacy (Table 3). Accordingly, we propose that Homeodynamic Rejuvenation is best assessed using controlled perturbation–recovery paradigms, consistent with principles established in hormesis research (Calabrese, 2008; Mattson, 2008). For example, controlled perturbation challenges, such as UV exposure or barrier disruption, could be used to quantify recovery kinetics and assess homeodynamic plasticity across domains.

**TABLE 3:** Clinical and cellular biomarkers of homeodynamic plasticity.

Domain	Description	Cellular biomarker	Clinical validation metric
Intracellular quality control	Maintenance of proteome integrity, organelle turnover, and redox balance through coordinated quality control systems	DNA repair (CPD △ and 8-oxoG △); proteostasis pathways △; autophagy markers △; ROS ○; antioxidant enzymes (CAT, SOD, GSR, and GST) ○; GSH:GSSG ratio △; lipid peroxidation ○; protein carbonylation △	Visible oxidative tone •; skin dullness •; total antioxidant capacity (tape strips) ○; sebum oxidation (squalene peroxides) ○
Regenerative competence	Preservation of the ability to repair, renew, and regenerate tissue following damage	Keratinocyte proliferation △; fibroblast activity △; collagen I synthesis △; Ki67 △; stem cell function markers △	Epidermal turnover rate ○; barrier recovery rate •; TEWL recovery half-time (t½) •; erythema resolution kinetics •; wound closure rate •
Metabolic resilience	Capacity to sustain energy production, redox equilibrium, and metabolic adaptability under stress	PPARGC1A (PGC-1α) △; mitochondrial membrane potential △; ATP △; NAD^+^/NADH ratio △	Microcirculation/perfusion ○; oxygenation (StO_2_) ○; reduction in dullness/grayness (b*) •
Cellular integrity	Regulation of intercellular signaling, inflammatory balance, and maintenance of cellular function	p16^INK4a^ △; p21^CIP1^ △; SASP cytokines (IL-6, IL-8, and TNF-α) ○	Baseline erythema (a*) •; recovery from repeated perturbation •; skin tone heterogeneity (ΔE) •
Structural integrity	Maintenance of extracellular matrix composition, mechanical properties, and tissue architecture	Collagen I/III ratio △; elastin △; fibronectin △; MMP-1/3 △; TIMP-1 △; basement membrane cohesion △	Viscoelasticity (R2 and R5) •; skin firmness (R0 and R7) •; wrinkle depth/volume •; dermal density/echogenicity ○

Legend: • clinically accessible (non-invasive); ○ translational/advanced; △ research-level (biopsy, *ex vivo*, or specialized infrastructure).

Abbreviations: 8-oxoG, 8-oxo-7,8-dihydroguanine; AGE, advanced glycation end-product; AhR, aryl hydrocarbon receptor; ATP, adenosine triphosphate; CAT, catalase; CPD, cyclobutane pyrimidine dimer; GSH, reduced glutathione; GSR, glutathione reductase; GSSG, oxidized glutathione; GST, glutathione S-transferase; IL-6, interleukin-6; IL-8, interleukin-8; Ki-67, proliferation marker; MMP-1, matrix metalloproteinase-1; MMP-3, matrix metalloproteinase-3; NAD^+^/NADH, nicotinamide adenine dinucleotide (oxidized/reduced); Nrf2, nuclear factor erythroid 2-related factor 2; PGC-1α (PPARGC1A), peroxisome proliferator-activated receptor gamma coactivator 1-alpha; p16^INK4a^, cyclin-dependent kinase inhibitor; p21^CIP1^, cyclin-dependent kinase inhibitor; ROS, reactive oxygen species; SASP, senescence-associated secretory phenotype; SOD, superoxide dismutase; TIMP-1, tissue inhibitor of metalloproteinases-1; TNF-α, tumor necrosis factor alpha.

**FIGURE 4:**
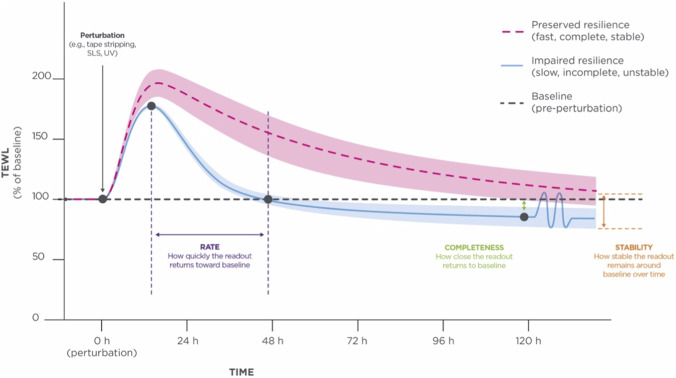
Conceptual model of controlled perturbation–recovery dynamics to measure homeodynamic plasticity. Schematic illustration of a hypothetical perturbation–recovery response (e.g., transepidermal water loss, TEWL) following an acute insult [e.g., tape stripping, sodium lauryl sulfate exposure, or UV irradiation]. The resulting trajectory defines three core dimensions of resilience, namely, rate, completeness, and stability.

Within this framework, resilience can be quantified along three measurable dimensions, namely, the rate of recovery, the completeness of return to baseline, and the stability of responses under repeated challenge (Figure 4). These parameters are, in principle, quantifiable across multiple biological domains, including barrier function, DNA repair, and redox homeostasis. Interventions consistent with restoration of the skin’s homeodynamic plasticity are expected to accelerate recovery following controlled perturbation, improve the completeness of restoration toward baseline function, and enhance the consistency of responses under repeated stress exposure. Moreover, improvements in these recovery parameters are also hypothesized to correlate with changes in clinically relevant markers of photoaging (Table 3).

In facial skin, for example, sequential tape stripping has been shown to increase transepidermal water loss (TEWL) by approximately twofold above baseline, with partial recovery detectable within 24 h (Gorcea et al., 2013). Thus, for barrier disruption, the rate can be expressed as the slope of TEWL normalization or the time required to reduce excess TEWL by 50%; completeness as the residual elevation in TEWL at defined time points (e.g., 24 h, 72 h, or 7 days); and stability as the consistency of recovery trajectories following repeated standardized challenges within the same individual (Figure 4). At present, no diagnostic cutoffs for “preserved” versus “impaired” resilience exist, but it can be estimated based on the existing literature (Törmä et al., 2008; Gorcea et al., 2013). As a provisional empirical framework, preserved barrier recovery could be defined by a substantial (i.e., ≥40–60%) decrease in excess TEWL within the first 24 h and return to baseline within several days, whereas impaired recovery would be indicated by limited improvement (i.e., <20–30%) at 24 h and persistent barrier abnormality over a multiday window.

For UV perturbation, analogous metrics can be derived from erythema decay curves, recovery of baseline colorimetric indices, or normalization of selected molecular damage-response markers.

Following controlled UVB exposure, erythema typically develops within 3–5 h, peaks at approximately 12–24 h, and usually resolves by approximately 72 h, providing a well-established time window over which recovery can be quantified (Gilchrest et al., 1981). Recovery could, thus, be defined as erythema that follows the expected rise-and-resolution pattern over 72 h, whereas impaired recovery would involve delayed resolution or persistent inflammation beyond that period.

This dynamic perspective distinguishes Homeodynamic Rejuvenation from static damage-accumulation models. Within this paradigm, youthfulness is not solely defined by low baseline damage but by preserved recovery capacity and the ability to maintain functional equilibrium under repeated environmental stress.

## 4 Conclusion

Homeodynamic Rejuvenation is defined not as the reversal of visible signs of aging but rather as the restoration of functional competence and biological vitality. It is grounded in the understanding that skin aging is no longer understood as a simple accumulation of damage but rather the progressive erosion of the skin’s capacity to recover and re-establish equilibrium within a changing environment. Accordingly, Homeodynamic Rejuvenation reframes intervention as one of restoration rather than repair, emphasizing reactivation of the skin’s intrinsic systems of adaptation, renewal, and balance.

By integrating principles of homeodynamics with exposome biology, Homeodynamic Rejuvenation translates this concept into measurable biological and clinical domains, providing a structured basis for quantifying and restoring skin’s adaptive capacity.

Taken together, this perspective establishes a mechanistically grounded approach with potential clinical relevance to skin aging. By targeting a limited set of biological domains, it becomes possible to influence a broad range of downstream molecular, cellular, and structural processes that collectively shape the visible trajectory of skin aging.

## Data Availability

The original contributions presented in the study are included in the article/supplementary material, further inquiries can be directed to the corresponding author.
